# Activation of the Constitutive Androstane Receptor Inhibits Leukocyte Adhesiveness to Dysfunctional Endothelium

**DOI:** 10.3390/ijms22179267

**Published:** 2021-08-27

**Authors:** Mireia López-Riera, Rebeca Ortega, Luisa Hueso, María Carmen Montesinos, Mari Carmen Gomez-Cabrera, María Jesús Sanz, José T. Real, Laura Piqueras

**Affiliations:** 1 Institute of Health Research-INCLIVA, 46010 Valencia, Spain; mireia.lopez.riera@gmail.com (M.L.-R.); rebeca.ortegaherraiz@gmail.com (R.O.); lmhueso.biotech@gmail.com (L.H.); carmen.gomez@uv.es (M.C.G.-C.); maria.j.sanz@uv.es (M.J.S.); Jose.T.Real@uv.es (J.T.R.); 2 Department of Pharmacology, University of Valencia, 46010 Valencia, Spain; m.carmen.montesinos@uv.es; 3 Center of Molecular Recognition and Technological Development (IDM), 46100 Burjassot, Spain; 4 Endocrinology and Nutrition Service, University Clinic Hospital of Valencia, 46010 Valencia, Spain; 5 Biomedical Network Research Centre in Diabetes and Associated Metabolic Diseases (CIBERDEM), 28029 Madrid, Spain; 6 Freshage Research Group, Department of Physiology, Faculty of Medicine, University of Valencia, 46010 Valencia, Spain; 7 Biomedical Network Research Centre on Frailty and Healthy Aging (CIBERFES), 28029 Madrid, Spain

**Keywords:** constitutive androstane receptor, leukocyte recruitment, endothelial dysfunction

## Abstract

Leukocyte cell recruitment into the vascular subendothelium constitutes an early event in the atherogenic process. As the effect of the constitutive androstane receptor (CAR) on leukocyte recruitment and endothelial dysfunction is poorly understood, this study investigated whether the role of CAR activation can affect this response and the underlying mechanisms involved. Under physiological flow conditions, TNFα-induced endothelial adhesion of human leukocyte cells was concentration-dependently inhibited by preincubation of human umbilical arterial endothelial cells with the selective human CAR ligand CITCO. CAR agonism also prevented TNFα induced VCAM-1 expression, as well as MCP-1/CCL-2 and RANTES/CCL-5 release in endothelial cells. Suppression of CAR expression with a small interfering RNA abrogated the inhibitory effects of CITCO on these responses. Furthermore, CITCO increased interaction of CAR with Retinoid X Receptor (RXR) and reduced TNFα-induced p38-MAPK/NF-κB activation. In vivo, using intravital microscopy in the mouse cremasteric microcirculation treatment with the selective mouse CAR ligand TCPOBOP inhibited TNFα-induced leukocyte rolling flux, adhesion, and emigration and decreased VCAM-1 in endothelium. These results reveal that CAR agonists can inhibit the initial inflammatory response that precedes the atherogenic process by targeting different steps in the leukocyte recruitment cascade. Therefore, CAR agonists may constitute a new therapeutic tool in controlling cardiovascular disease-associated inflammatory processes.

## 1. Introduction

Atherosclerosis involves an inflammatory response, consisting of intimal accumulation of leukocytes and lipid-laden macrophages throughout the entire atherogenic process [1,2]. Endothelial dysfunction, one of the earliest stages of atherogenesis, leads to a prothrombotic and proinflammatory phenotype of the endothelium which induces leukocyte attachment and subsequent migration [3]. Expression of diverse cell adhesion molecules and secretion of several proinflammatory cytokines and chemokines by immune and endothelial cells also contribute to the leukocyte infiltration process [4,5].

The constitutive androstane receptor (CAR) is an important member of the nuclear receptor superfamily (subfamily 1, group I, member 3 [NR1i3]), which regulates diverse aspects of organ physiology [6,7]. Initially, CAR was described as a xenosensor that mediates xenobiotic drug-induced changes by increasing transcription of genes involved in drug clearance and disposition; however, later studies demonstrated an alternative role for CAR in regulating hormone levels in response to metabolic and nutritional stress [6]. The discovery of its structure and ligands allowed exploration of the role of CAR in several diseases, particularly cancers [6,8], Crohn’s disease [9], obesity [10] and type 2 diabetes [11]. Nonetheless, the role of CAR in other cardiovascular-related conditions such as endothelial dysfunction remain largely unknown.

With the aim of identifying more effective strategies to treat and prevent endothelial dysfunction associated with pathologies such as atherosclerosis, and co-morbid metabolic disorders characterized by endothelial dysfunction, herein, we evaluate the effect of CAR agonism in leucocyte-endothelial cell attachment. In this study, using several experimental approaches, we provide in vitro evidence in human vascular cells and in vivo in mouse cremasteric microcirculation that CAR activation results in clear impairment in TNFα-induced leukocyte cell adhesion to the endothelium. We also explore the underlying mechanisms involved in these responses.

## 2. Results

### 2.1. CAR Is Expressed in Human Endothelial Cells

**Figure 1:**
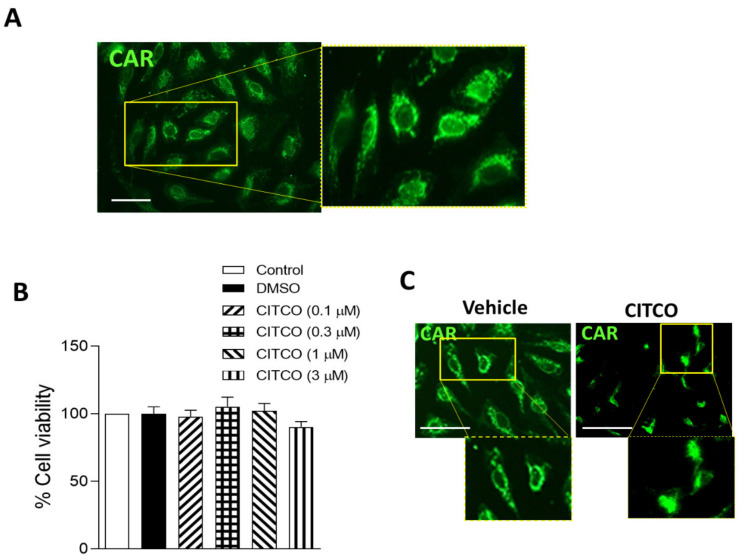
CITCO induces CAR translocation into the nucleus in human vein endothelial cells (HUVEC). (**A**) Cytoplasmic location of CAR detected by immunofluorescence analysis in endothelial cells. Scale bar = 50 µm. (**B**) Effect of CITCO (0.1–3 µM) on endothelial cell viability. Data are presented as mean ± SEM of *n* = 5 independent experiments. (**C**) Representative images showing CAR translocation from the cytoplasm into the nucleus after CITCO (3 µM) treatment in HUVEC. Immunoreactivity was visualized using an Alexa Fluor 488 secondary antibody (CAR, green). Scale bar = 50 µm.

First, CAR expression was analyzed in human umbilical vein endothelial cells (HUVEC). As Figure 1A shows, CAR protein expression was detected by immunofluorescence analysis, and we observed that CAR was mainly localized in the cytoplasm compartment of endothelial cells (Figure 1A). We next studied the effect of CAR-selective agonist 6-(4-chlorophenyl) imidazo [2,1-b][1,3]thiazole-5-carbaldehyde O-(3,4-dichlorobenzyl)oxime (CITCO) on endothelial cell viability. For this purpose, HUVEC monolayers were pretreated with CITCO (0.1–3 μM) or vehicle (0.1% DMSO) for 24 h. As illustrated in Figure 1B, no effects on endothelial cell viability were detected at the different CITCO concentrations used. Interestingly, immunofluorescence assays revealed that while in vehicle-treated cells, CAR receptor remains in the cytoplasmic compartment of endothelial cells, after CITCO treatment, CAR translocated from the cytoplasm into the nucleus (Figure 1C).

### 2.2. CITCO Inhibits TNFα-Induced Leukocyte–Endothelial Cell Interactions under Flow Conditions

**Figure 2:**
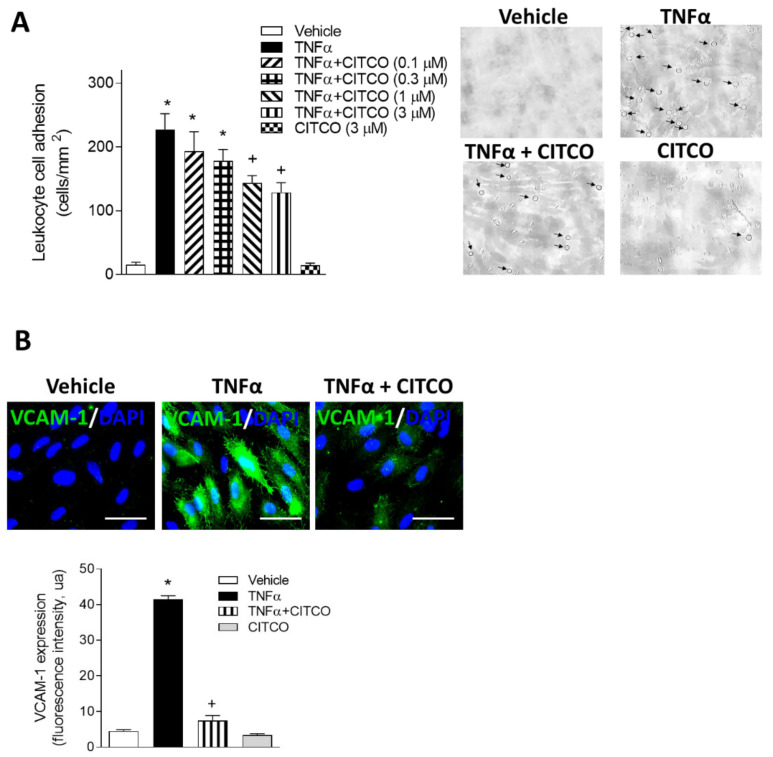
Effect of CITCO on leukocyte–endothelial cell interactions under physiological flow conditions. (**A**) HUVEC were treated with vehicle (0.1% DMSO) or CITCO (0.1–3 μM) for 24 h and then stimulated with TNFα (20 ng/mL, 24 h). Next, whole blood from healthy volunteers was perfused across the endothelial monolayers for 5 min at 0.5 dynes/cm^2^, and leukocyte adhesion was quantified. Data are mean ± SEM (*n* = 8 for each group). * *p* < 0.05 relative to vehicle group, + *p* < 0.05 relative to TNFα stimulated cells. Representative images of leukocyte adherence to HUVEC monolayers under different treatment conditions are also shown. Arrows indicate adhered cells. (**B**) Effect of CITCO on VCAM-1 expression. Cells were pretreated with vehicle (0.1% DMSO) or CITCO (3 µM) 24 h before TNFα stimulation (20 ng/mL, 24 h). Representative images of VCAM-1 expression in HUVEC are shown. Immunoreactivity was visualized using an Alexa Fluor 488 secondary antibody (VCAM-1, green). Nuclei were counterstained with DAPI (blue). Scale bar = 50 µm. Values are expressed as mean ± SEM, *n* = 3. * *p* < 0.05 relative to vehicle group, + *p* < 0.05 relative to TNFα-stimulated cells.

Next, we evaluated the effect of CAR activation on leukocyte–endothelial cell interactions in vitro using the dynamic flow chamber assay. For this purpose, freshly drawn human whole blood from healthy volunteers was perfused across HUVEC monolayers stimulated or not with TNFα (20 ng/mL) for 24 h. As expected, TNFα caused a significant increase in leukocyte adhesion to endothelial cells compared to vehicle-treated cells (*p* < 0.05, Figure 2A). To determine the effects of CAR agonist CITCO on TNFα-induced leukocyte cell recruitment, HUVEC were pretreated with CITCO (0.1–3 μM) prior to TNFα stimulation. Significant reductions in TNFα-induced leukocyte adhesion were observed in a concentration-dependent manner (Figure 2A). Additional immunofluorescence assays were performed to investigate whether the inhibitory effects exerted by CAR agonism on adhesion were mediated by modulating the cellular adhesion molecule VCAM-1 (Figure 2B). Stimulation with TNFα resulted in clear upregulation of VCAM-1 compared with unstimulated control HUVEC (*p* < 0.05, Figure 2B). Pretreatment of cells with CITCO (3 μM) resulted in a decrease in TNFα-induced VCAM-1 expression (*p* < 0.05, Figure 2B).

### 2.3. CAR Silencing by siRNA Blocks the Anti-Inflammatory Effect of CITCO in Endothelial Cells

**Figure 3:**
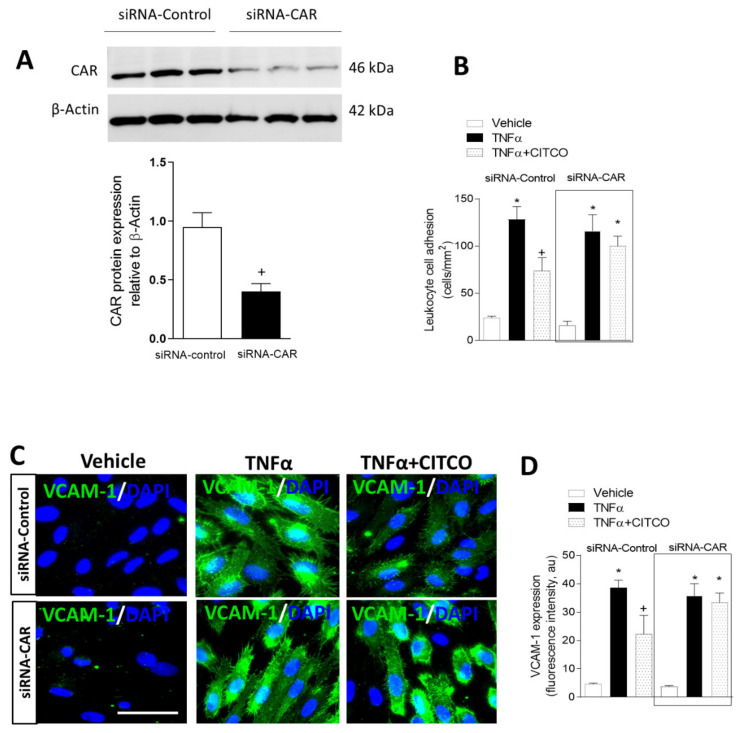
Knockdown of CAR by siRNA blocks the suppressive effect of CITCO on TNFα-induced leukocyte–endothelial interactions and VCAM-1 expression. Endothelial cells were transfected with control or CAR-specific siRNA. At 48 h post-transfection, cells were pretreated with CITCO (3 µM) for 24 h and then stimulated with TNFα (20 ng/mL, 24 h). (**A**) CAR protein expression in transfected HUVEC was analyzed by Western blot. Protein quantification was performed by densitometry and CAR protein levels were normalized to β-actin. Values are expressed as mean ± SEM (*n* = 6). +*p* < 0.05 relative to control siRNA transfected cells. (**B**) After transfection HUVEC were treated with vehicle (0.1% DMSO) or CITCO (3 μM) for 24 h and then stimulated with TNFα (20 ng/mL, 24 h). Next, whole blood from healthy volunteers was perfused across the endothelial monolayers for 5 min at 0.5 dynes/cm^2^ and leukocyte adhesion was quantified. Data are mean ± SEM (*n* = 4 for each group). * *p* < 0.05 relative to the respective vehicle group, + *p* < 0.05 relative to the respective TNFα-stimulated cells. (**C**) Representative images showing VCAM-1 expression in transfected HUVEC. Immunoreactivity was visualized using an Alexa Fluor 488 secondary antibody (VCAM-1, green). Nuclei were counterstained with DAPI (blue). Scale bar = 50 µm. (**D**) Fluorescence intensity of Alexa Fluor 488 (VCAM-1, green) was quantified using ImageJ. Values are expressed as mean ± SEM, *n* = 6. * *p* < 0.05 relative to the respective vehicle group, + *p* < 0.05 relative to the respective TNFα-stimulated cells.

To gain further insight into the underlying mechanisms of CAR agonism, we next investigated the potential involvement of CAR in the effects of CITCO, performing CAR-specific silencing assays for this purpose. Forty-eight hours post-transfection with a CAR-specific siRNA, HUVEC showed a ~70% reduction in CAR protein levels compared with control siRNA-transfected cells (*p* < 0.05, Figure 3A). Interestingly, CAR-specific siRNA abolished the suppressive effects of CITCO on leukocyte–endothelial cell interactions (Figure 3B) and VCAM-1 expression induced by TNFα (Figure 3C,D).

**Figure 4:**
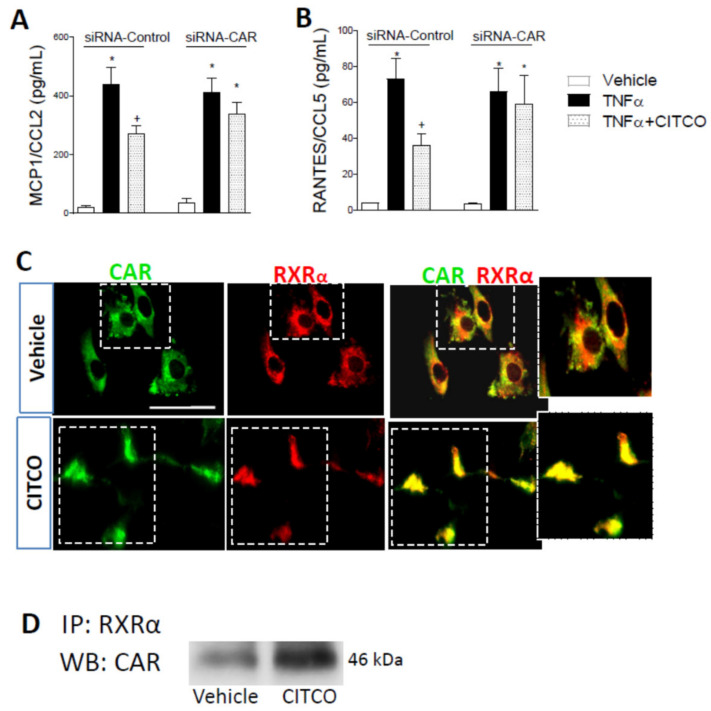
CITCO treatment reduced chemokine secretion in HUVEC. Endothelial cells were transfected with control or CAR-specific siRNA. At 48 h post-transfection, cells were pretreated with CITCO (3 µM) for 24 h and then stimulated with TNFα (20 ng/mL, 24 h). (**A**) MCP-1/CCL-2 and (**B**) RANTES/CCL5 release was determined by ELISA in the cell-free supernatants of transfected HUVEC (values were expressed in pg/mL). Data represent mean ± SEM (*n* = 5 independent experiments). * *p* < 0.05 relative to the respective vehicle group, + *p* < 0.05 relative to the respective TNFα-stimulated cells. (**C**) CITCO promotes CAR/RXRα interaction in HUVEC. Representative images of CAR and RXRα and after CITCO treatment. Immunoreactivity was visualized using Alexa Fluor 488 (CAR, green) and Alexa Fluor 594 (RXR, red) secondary antibodies. Bar = 50 µm. (**D**) CAR/RXRα-interaction was assessed by immunoprecipitation. A representative blot is shown of *n* = 3 independent experiments. IP: immunoprecipitation. WB: Western Blot.

Given that TNFα stimulation of endothelial cells can trigger the release of different chemokines that contribute to leukocyte arrest, we next evaluated the effect of CAR agonist in TNFα-induced MCP-1/CCL2 and RANTES/CCL5 release from human endothelial cells. Significant increases in MCP-1/CCL2 and RANTES/CCL5 levels were detected in the supernatant of HUVEC stimulated with TNFα for 24 h. When HUVEC were transfected with control siRNA, this effect was inhibited by pretreating the cells with the CAR ligand CITCO at 3 µM (Figure 4A,B). Conversely, when HUVECs were transfected with CAR siRNA, CITCO did not reduce chemokine release induced by TNFα (Figure 4A,B).

Ligand binding of CAR promotes heterodimerization with the RXRα receptor, inducing CAR transactivation of target genes [12,13], so we next explored the potential interaction of CAR with RXRα in response to CITCO treatment. By immunofluorescence assays, we found both CAR and RXRα in the cytoplasm of endothelial cells. However, when HUVEC are exposed to CITCO, both receptors translocate and colocalize in the nucleus of endothelial cells (Figure 4C). To corroborate these observations, immunoprecipitation assays were carried out to detect protein–protein interaction. As shown in Figure 4D, immunoprecipitation analysis revealed clear interaction between CAR and RXRα in human endothelial cells treated with CITCO.

### 2.4. CAR Activation Inhibits p38 MAPK and NF-κB and Signalling Pathways

**Figure 5:**
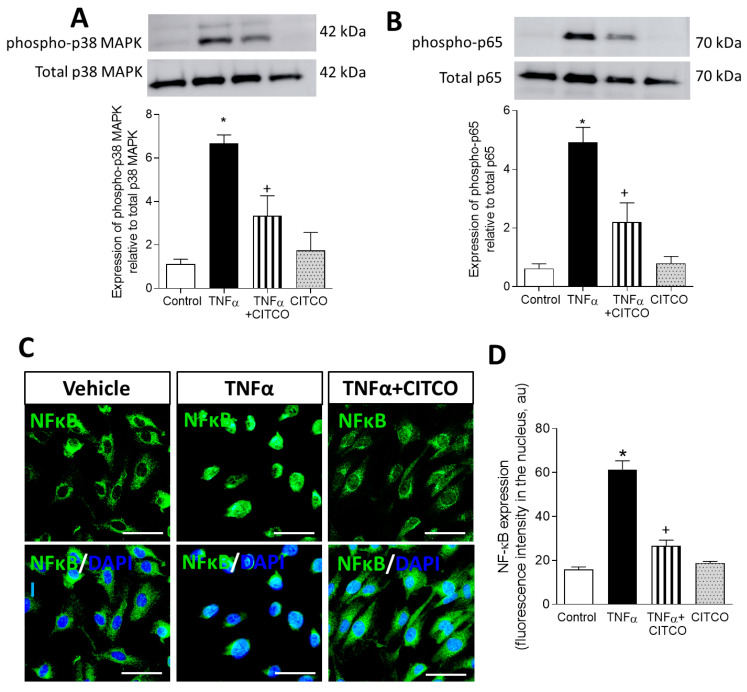
CITCO treatment diminishes p38 MAPK and p65 NF-κB phosphorylation induced by TNFα in HUVEC. Cells were pretreated with vehicle (0.1% DMSO) or CITCO (3 µM) for 24h before stimulation with TNFα (20 ng/mL) for 30 min. Representative Western blots of (**A**) phospho p38/total p38 MAPK and (**B**) phospho p65/total p65 in HUVEC subjected to the different treatments. Data represent mean ± SEM of protein densitometry (*n* = 5 for each group). * *p* < 0.05 relative to vehicle group, + *p* < 0.05 relative to TNFα stimulated cells. (**C**) Representative images of NF-κB translocation into nuclei after CITCO treatment. Immunoreactivity was visualized using Alexa Fluor 488 (NFκB, green) secondary antibody. Nuclei were stained with DAPI (blue). Scale bar = 50 µm. (**D**) Fluorescence intensity was quantified using ImageJ. Values are expressed as mean ± SEM (*n* = 3 for each group). * *p* < 0.05 relative to vehicle group, + *p* < 0.05 relative to TNFα stimulated cells.

The p38 MAPK and NF-kB signalling cascade has a dominant role in mediating TNFα-induced cellular adhesion molecular expression and chemokine release [13,14,15]; therefore, we next explored the effect of CAR activation on these intracellular pathways. By Western blot assays, we observed that exposure of HUVEC to TNFα produced a significant phosphorylation of p38 MAPK and p65 NF-kB, which was notably reduced when HUVEC were pretreated with CITCO (*p* < 0.05, Figure 5A,B). Furthermore, in additional immunofluorescence assays, CITCO pretreatment undermined the nuclear translocation of NF-kB induced by TNFα (*p* < 0.05, Figure 5C,D).

### 2.5. CAR Agonism Inhibits Leukocyte–Endothelial Cell Interactions In Vivo

**Figure 6:**
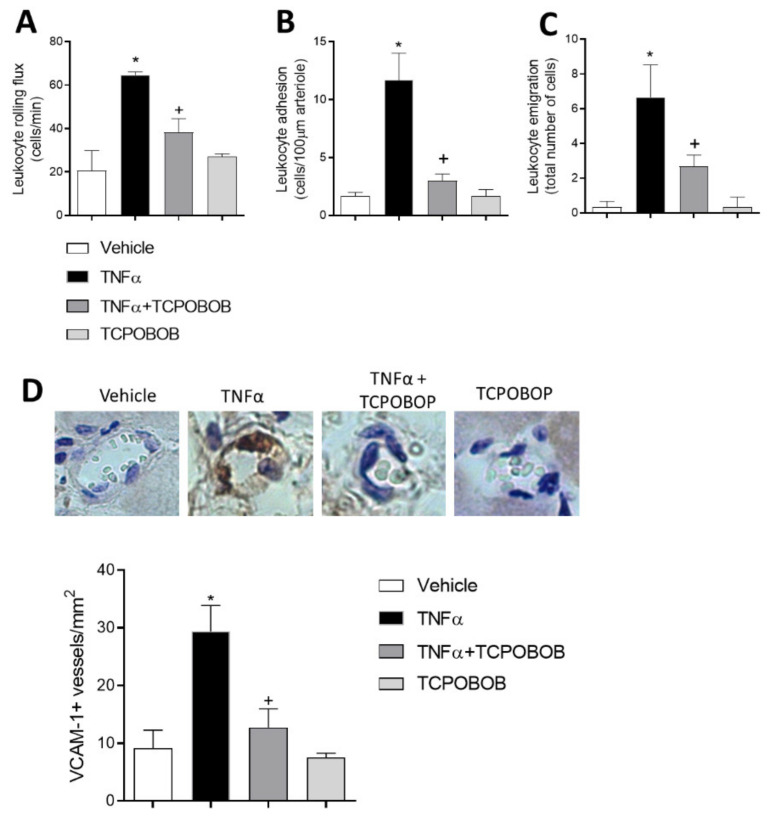
CAR activation inhibits leukocyte recruitment in murine cremasteric microvessels. Mice were treated for 24 h with TCPOBOP (3 mg/kg, i.p) or vehicle prior to TNF-α stimulation (0.5 µg/mouse, intrascrotal injection, 4 h) and leukocyte rolling (**A**), adhesion (**B**) and emigration (**C**). The results represent five experiments with each treatment. Some cremasters were fixed for microvessel staining with anti-VCAM-1 antibody. Immunolocalization of VCAM-1 on the vascular endothelium (**D**). Results are representative of three experiments with each treatment. * *p* < 0.05 relative to the vehicle group, + *p* < 0.05 relative to the TNFα-treated group.

To extend the functional data obtained in vitro to an in vivo model, intravital microscopy was used to examine leukocyte trafficking in mouse cremasteric microcirculation. In animal postcapillary venules, 4 h exposure to TNFα also induced a significant increase in venular leukocyte rolling flux, adhesion, and emigration (Figure 6A–C). Notably, pretreatment with selective mouse CAR agonist TCPOBOP significantly reduced TNFα-induced leukocyte–endothelial cell interactions (*p* < 0.05, Figure 6). Administration of TCPOBOP alone did not affect basal responses. Finally, immunofluorescence analysis of the cremasteric microvasculature showed that stimulation with this cytokine caused a substantial increase in VCAM-1 expression within the cremasteric postcapillary venules. Pretreatment of mice with TCPOBOP decreased TNFα-induced VCAM-1 expression (Figure 6D).

## 3. Discussion

Early studies described CAR as a xenobiotic sensor that regulates genes involved in detoxification and elimination of potentially harmful exogenous molecules. More recently, a number of experimental studies have reported beneficial effects of CAR agonism in type II diabetes and obesity by improving glucose tolerance, insulin sensitivity, inducing β-oxidation and reducing lipid deposition [11,16]. However, little is known about whether CAR can regulate metabolic syndrome-related vascular inflammation. In the current study, we have demonstrated, for the first time, that CAR operates in endothelial cells and its activation inhibits leukocyte recruitment to vascular dysfunctional endothelium.

Dysfunctional endothelium leads to increased vascular permeability which promotes leukocyte extravasation from the bloodstream to an inflammatory focus. To evaluate the influence of CITCO on leukocyte–endothelial cell interactions with human cells, we investigated the effect of CAR activation in an in vitro human system under flow conditions, trying to mimic the physiological events that occur in inflamed microcirculation. We found that CAR activation inhibited TNFα-induced leukocyte adhesion on activated HUVEC. It is well known that TNFα exerts potent proinflammatory actions in metabolic diseases such as obesity [17,18,19] and insulin resistance [20,21], with both disorders associated with high risk of developing cardiovascular alterations [22]. Elevated concentrations of circulating TNFα are linked with increased susceptibility to heart failure [23] and impaired glucose and lipid homeostasis [24] and this also contributes to atherosclerotic plaque thickening [25]. Counteracting TNFα-mediated effects has proven to ameliorate endothelial dysfunction and vascular conditions [26,27].

The decline in adhesive interactions induced by CAR agonism on leukocyte adhesion is suggestive of reduced adhesion molecule expression and/or function. Our results suggest that in human endothelial cells, CAR agonism inhibits TNFα-induced VCAM-1 expression, and also that CAR could operate as a heterodimer with the retinoid X receptor RXRα to modulate CAM expression. The importance of specific adhesion molecules in inflammatory cell recruitment to areas of inflammation is widely accepted. Activation and expression of adhesion molecules enable leukocyte adhesion, conformational change and extravasation (emigration), which in turn induces local injury and can help orchestrate subsequent systemic inflammation and its consequences [28]. Furthermore, we found that cell pretreatment with CAR but not control siRNA blunted the inhibitory response elicited by CAR on TNF-α-induced VCAM-1 expression.

Little is known regarding the impact of CAR activation on human chemokine release. It is well established that vascular endothelial cell activation with TNFα triggers production of a wide array of leukocyte-recruiting chemokines. Among these, MCP-1/CCL2 and RANTES/CCL5 have been shown to play a critical role in mononuclear leukocyte arrest and participate in the progression of atherosclerotic lesions [29,30]. In the present study, CITCO inhibited TNFα-elicited MCP-1/CCL2 and RANTES/CCL5 release, an effect blunted in cells lacking CAR, suggesting that CAR activation decreases leukocyte arrest by inhibiting chemokine generation and release. CAR activation has been reported to reduce inflammatory response in several pathologic conditions. In this way, CAR protected against experimental colitis by inhibiting proinflammatory cytokines [31]. In that study, Uehara et al. showed that NF-κB target genes such as IL-1β and chemokine receptor CCR2 were markedly down-regulated by CAR agonism [31].

The dominant role of the p38 MAPK/NF-κB signalling pathway in mediating TNFα-induced cytokine/chemokine synthesis, endothelial cell dysfunction and cellular molecular adhesion expression in atherosclerosis has been convincingly demonstrated [32,33,34]. Based on our findings, it is tempting to speculate that CAR agonism decreases those inflammatory mediators through the NF-kB signalling pathway in dysfunctional endothelium. However, other possible molecular pathways could be involved in the anti-inflammatory effects of CAR activation. In our study, we have observed a heterodimerization of CAR with RXRα; therefore, that complex could directly bind to specific element responses to DNA and transactivate target genes. Whether or not CAR could modulate other signalling pathways remains an open question and further investigation is warranted to elucidate the impact of CAR activation in vascular diseases.

Whereas CITCO is a selective CAR activator in humans, TCPOBOP is a potent and selective CAR activator in mice [35,36]. In the current study, using intravital microscopy to directly assess the effect of CAR activation on leukocyte–endothelial cell interactions, we have shown that in vivo TCPOBOP is also capable of inhibiting TNFα-induced VCAM-1 expression in the postcapillary venules of the cremasteric microcirculation. Along these lines, recent studies have suggested a role for CAR as a potential target in atherosclerosis and hypercholesterolemia [37]. Thus, the CAR agonist TCPOBOP decreased plasma TG-rich lipoprotein and intermediate-density lipoprotein (IDL)/LDL levels, resulting in a significant reduction in the size of atherosclerotic lesions in the aortic valves of Ldlr−/− mice [37]. Furthermore, in murine models of obesity induced by high fat diet, TCPOBOP showed beneficial effects by improving insulin sensitivity and hepatic steatosis [11]. In this study, the metabolic improvement of CAR activation was associated with the combined effect of lipogenesis and gluconeogenesis inhibition as well as increased brown adipose tissue energy expenditure and peripheral fat mobilization [11]. Regarding the anti-inflammatory role of CAR, a previous transcriptome study in mouse liver carried out by Cui et al. [38] showed that TCPOBOP treatment down-regulated several genes critically involved in inflammatory response, such as chemokine (C-X-C motif) ligand 1 (Cxcl1), IL1α and IFNα, but also genes important in intermediary metabolism, including glucose-6-phosphatase [38]. Similarly, CAR activation by TCPOBOP reduced cholestasis-induced liver dysfunction, inflammation (p65 nuclear translocation, mRNA expression of TNFα, IL-1β, IL-6) and oxidative stress [39].

**Figure 7:**
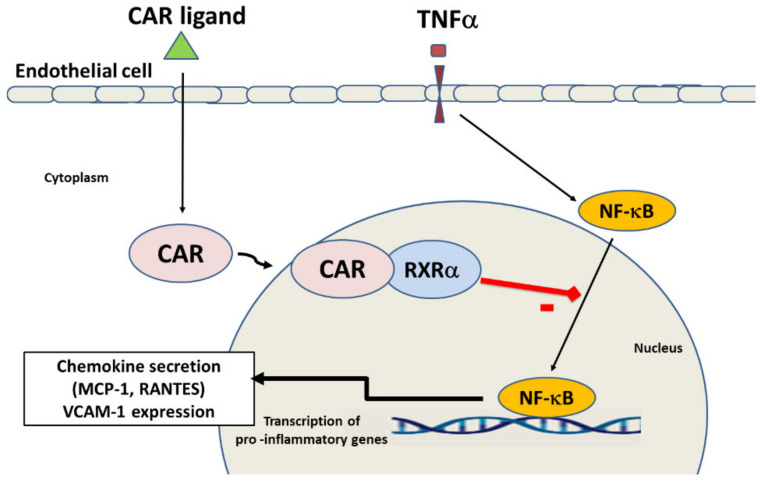
Graphical abstract showing the effects of CAR activation in endothelial cells. Activation of CAR by a ligand, promotes its translocation from the cytoplasm into the nucleus and heterodimerization with RXRα. This complex inhibits NF-κB activation induced by TNFα and consequently the transcription of the proinflammatory genes MCP-1, RANTES and VCAM-1 in endothelial cells. Black lines mean activation. Red line with rectangle means inhibition.

A schematic diagram illustrating the potential targets of CAR to inhibit inflammatory response and endothelial dysfunction is shown in Figure 7. Briefly, activation of CAR receptor by a ligand agonist, induces its nuclear translocation and heterodimerization with RXRα and inhibits TNFα induced production of inflammatory mediators including RANTES, MCP-1 and VCAM-1 expression in endothelial cells through suppression of the NF-κB pathway.

## 4. Material and Methods

### 4.1. Endothelial Cell Culture

Human umbilical vein endothelial cells (HUVEC) were isolated by collagenase treatment [40] and maintained in human endothelial cell basal medium-2 (EBM-2, Lonza, Barcelona, Spain) supplemented with endothelial growth factor medium-2 (EGM-2, Lonza, Barcelona, Spain) and 10% FBS. Cells up to passage one were grown to confluence on 24-well culture plates. Prior to each experiment, cells were incubated 16 h in medium containing 1% FCS and then returned to the 10% FCS medium at the beginning of all experimental protocols.

### 4.2. Viability Assay

HUVEC (2 × 10^5^ cells/well) were seeded in 96-well plates and treated with the CAR agonist O-[(3,4-dichlorophenyl)methyl]oxime 6-(4-chlorophenyl)-imidazo[2,1-b]thiazole-5-carboxaldehyde (CITCO) (0.1–3 μM) (Cayman Chemical, Ann Arbor, MI, USA) or vehicle (0.1% Dimethyl sulfoxide, DMSO) for 24 h at 37 °C, after which cells were added with 20 µL of 3-[4,5-dimethylthiazole-2-yl]-2,5-diphenyltetrazolium bromide (MTT) solution (5 g/L) (Sigma-Aldrich, Saint Louis, MO, USA) for 4 h incubation. Next, the supernatant was discarded, and the precipitate was dissolved, followed by adding 200 µL dimethyl sulfoxide. The optical density value of each well was measured at 490 nm using a spectrophotometer (Infinite M200, Tecan, Mannedorf, Switzerland). The values of each group were normalized to those of untreated HUVEC.

### 4.3. Leukocyte–HUVEC Interactions under Flow Conditions

HUVEC were grown to confluence and stimulated with TNFα (20 ng/mL)(PreproTech, London, UK) for 24 h. Before TNFα treatment, endothelial cells were preincubated with CITCO (0.1–3 µM) or vehicle (0.1% DMSO) for 24 h. Heparinized whole blood from healthy donors was diluted 1:10 in Hanks’ Balanced Salt Solution (HBSS) (Cat# 10-508F, Lonza, Barcelona, Spain). Next, the GlycoTech flow chamber was assembled and placed onto an inverted microscope stage, and freshly drawn human blood was then perfused across the endothelial monolayers. In all experiments, leukocyte adhesion was determined after 5 min at 0.5 dynes/cm^2^. Cells interacting on the surface of the endothelium were visualized and recorded (×20 objective, ×10 eyepiece) using phase-contrast microscopy (Axio Observer A1, Gottinger, Germany).

### 4.4. Chemokine Detection

The human chemokines MCP-1/CCL2 and RANTES/CCL5 were measured in HUVEC supernatants using antibody pairs from R&D Systems following the manufacturer’s instructions (ELISA Duoset kits, Abingdon, UK). Briefly, after coating the plates overnight with the primary antibodies, nonspecific binding sites were blocked with 3% BSA for 1 h. Supernatants and standards were added to PBS/1%/BSA/0.05% sodium azide for 2 h. Biotinylated detector antibodies were added for 2 h, followed by streptavidin-HRP for 1 h. All plate washes were carried out in four cycles with freshly prepared PBS/0.05% Tween20. Enhanced K-Blue tetramethylbenzidine substrate (Thermo Fisher Scientific, Waltham, MA, USA) was added for 30 min and the enzyme reaction was stopped by adding 0.19M sulfuric acid. Absorbance was read at 450 nm. The obtained chemokine concentration in the supernatants is expressed as pg/mL.

### 4.5. Immunofluorescence Analysis

Confluent HUVEC were grown on glass coverslips, some of which were treated for 24 h with CITCO (3 µM) or vehicle, and then stimulated for 24 h with TNFα (20 ng/mL). The cells were then washed with PBS, fixed with 4% paraformaldehyde and blocked in PBS/1% BSA solution. Protein localization was detected by indirect immunofluorescence using the following primary antibodies: rabbit monoclonal anti-human CAR (1:50 dilution, cat# ab186869, Abcam, Waltham, MA, USA), mouse monoclonal anti-human VCAM-1 (1:50 dilution, cat# MCA907, BioRad, Barcelona, Spain), mouse monoclonal anti-human phosphorylated-NFκB (1:100 dilution, cat# 610869, BD Biosciences, Madrid, Spain) and goat polyclonal anti-human RXRα (1:50 dilution, cat#ab24636, Abcam, Waltham, MA, USA). Immunofluorescence signals were detected using the following secondary antibodies: Alexa Fluor 488 goat anti-mouse (1:1000 dilution, cat# A11001), Alexa Fluor 488 goat anti-rabbit (1:1000 dilution, cat# A11034) or Alexa Fluor 594 chicken anti-goat (1:1000 dilution, cat#A21468), all from Molecular Probes (Life Technology, Eugene, OR, USA). To confirm specificity of antibodies, isotype controls (cat# 172730; cat# ab18451, respectively, both from Abcam, Waltham, MA, USA) or secondary antibodies only were used as negative controls. Nuclei were stained with 4′,6-diamidino 2-phenylindole (DAPI, Thermo Fisher Scientific, Waltham, MA, USA).

### 4.6. Western Blotting

Protein concentrations from endothelial cells extracts were determined by the BCA method (Pierce BCA Protein Assay, Thermo Fisher Scientific, Waltham, MA, USA). Samples were denatured, subjected to 12% SDS-PAGE and transferred to a nitrocellulose membrane. Non-specific binding sites were blocked with 5% BSA in TBS solution and membranes were incubated overnight with following primary rabbit polyclonal antibodies: CAR (dilution 1:500, cat# ab186869, Abcam Waltham, MA, USA); phosphorylated-p38 MAPK (dilution 1:500, Thr180/Tyr182, cat#9211, Cell Signalling, Beverly, MA, USA), p-38 MAPK (dilution 1:500, cat#9212, Cell Signaling, Beverly, MA, USA), phosphorylated-p65 (dilution 1:200, Ser536, cat# 3033, Cell Signalling, Beverly, MA, USA), NF-κB p65 (dilution 1:200, cat#4764, Cell Signalling, Beverly, MA, USA) and mouse monoclonal antibody against human β-actin (dilution 1:2000, cat# 3700, Cell Signalling, Beverly, MA, USA). Next, membranes were washed and incubated for 1 h with a secondary HRP-linked anti-rabbit antibody (dilution 1:2000, cat#0448, Dako, Glostrup, Denmark). The blots were developed using a chemiluminescence detection system. Signals were recorded using a luminescent analyzer (ImageQuant™ LAS 500, GE Healthcare, Uppsala, Sweden) and quantified with ImageJ software (NIH, https://imagej.nih.gov/ij/ (accessed on 22 July 2021)).

### 4.7. CAR Silencing via Small Interfering RNA

Confluent HUVEC were transfected with either control or CAR-specific small interfering RNA (siRNA; Dharmacon, Lafayette, CO, USA) using Lipofectamine RNAiMAX (Invitrogen, Carlsbad, CA, USA) for 48 h following manufacturers’ instructions. Thereafter, cells were treated for 24 h with CITCO 3 µM or vehicle, and were then stimulated for 24 h with TNFα (20 ng/mL).

### 4.8. Immunoprecipitation

HUVEC extracts were prepared in 25 mM Tris-HCl (pH 8), 150 mM NaCl, 1% Nonidet P-40, 1 mM EDTA, and a mixture of protease (1 mM PMSF, 40 μg.mL^−1^ aprotinin, and 40 μg.mL^−1^ leupeptin) and phosphatase (1 mM sodium orthovanadate and 1 mM NaF) inhibitors (all from Sigma Aldrich, Saint Louis, MO, USA). Protein (200 μg) was incubated with 5 μg of rabbit monoclonal antibody against RXRα (cat#sc553, Santa Cruz Biotechnology, Dallas, Texas, USA). Immunocomplexes were precipitated using anti-rabbit IgG beads (cat# 8800, eBioscience, San Diego, CA, USA) and suspended in sample buffer containing 50 mM dithiothreitol (DTT). Western blotting was performed with a rabbit monoclonal antibody against CAR (dilution 1:500, cat# ab186869, Abcam, Waltham, MA, USA). Membranes were incubated with the secondary HRP-linked antibody (1:2000 dilution, cat# 0448, Dako, Glostrup, Denmark) and chemiluminescent signals were developed with ECL (GE Healthcare, Uppsala, Sweden). Signals were recorded using a luminescent analyzer (ImageQuant™ LAS 500, GE Healthcare, Uppsala, Sweden).

### 4.9. Intravital Microscopy Studies

Protocols followed the European Union guidelines for animal care and protection and were approved by the Ethics Review Board of the Faculty of Medicine, University of Valencia (Spain). All efforts were made to minimize the number of animals used and their suffering. C57BL/6/129Sv male mice were supplied by Charles River Laboratories (Chatillon-sur-Chalaronne, France). Mice were housed under specific pathogen-free conditions with free access to a normal chow diet and water, at a constant temperature (22 ± 2 °C) and humidity (60–65%) with a 12 h dark/light cycle (lights on at 8:00 h).

Mice (C57BL/6/129Sv), 6 weeks of age, were used for intravital microscopy studies as previously described [41,42]. Mice were treated intraperitoneally with 1,4-bis((3,5-dichloropyridin-2-yl)oxy)benzene (TCPOBOP) (3 mg/kg) (Sigma Aldrich, Saint Louis, MO, USA) or with vehicle alone over a 24 h period. After this, the cremaster micro-circulation was stimulated with 0.5 mg TNFα in 0.3 mL isotonic saline by intrascrotal injection, and determinations were performed 4 h later. The TCPOBOP dose was within the range previously used in mice [37,43]. After treatments, animals were anesthetized by i.p. injection with a mixture of xylazine hydrochloride (10 mg/kg, Sigma Aldrich, Saint Louis, MO, USA) and ketamine hydrochloride (200 mg/kg, Sigma Aldrich, Saint Louis, MO, USA). The cremaster muscle was dissected free of tissues and exteriorized onto an optical clear-viewing pedestal. The muscle was cut longitudinally with a cautery and held flat against the pedestal by attaching silk sutures to the corners of the tissue. The muscle was then perfused continuously at a rate of 1 mL/min with warmed bicarbonate-buffered saline (pH 7.4).

An orthostatic microscope (Nikon Optiphot-2, SMZ1, Badhoevedorp, The Netherlands) equipped video camera (Sony SSC-C350P, Köln, Germany) was used to capture images. Single unbranched cremasteric venules (20–40 μm in diameter) were selected for study, and the diameter was measured on-line by using a video caliper (Microcirculation Research Institute, Texas A&M University, College Station, TX, USA). Rolling leukocytes were defined as those white blood cells moving more slowly than erythrocytes in the same vessel. Leukocyte rolling flux was measured as number of cells rolling past a fixed point in the vessel per min. The same reference point was used throughout, as leukocytes may roll for only a section of the vessel before rejoining the blood flow or becoming firmly adhered. A leukocyte was defined as adhered to venular endothelium if stationary for at least 30 s. Leukocyte adhesion was expressed as the number/100 μm length of venule. Leukocyte emigration was defined as the number of extravascular leukocytes visible per microscopic field centered on postcapillary venules, and was determined by averaging data derived from four to five fields.

### 4.10. Immunohistochemistry

After the intravital microscopy measurements were completed, the cremaster was isolated, fixed in 4% paraformaldehyde, dehydrated using graded acetone washes at 4 °C and embedded in paraffin wax for localization of VCAM-1 using a modified avidin and biotin immunoperoxidase technique, as previously described. Tissue sections (5 μm thick) were incubated for 1 h with antibody with anti-mouse VCAM-1 (dilution 1:50, BD Pharmingen, San Diego, CA, USA). Positive staining was defined as a venule displaying brown reaction product. VCAM-1 expression analysis was as previously described [41]; briefly, 20 venules were examined per group for VCAM-1 to determine the percentage of positively stained vessels.

### 4.11. Statistical Analysis

Values are expressed as means ± SEM. For comparisons of two groups, Student’s *t*-test was used in data that passed both normality (Kolmogorov–Smirnov test) and equal variance (Levene’s test); otherwise, a non-parametric Mann–Whitney *U* test was performed. For comparisons among multiple groups, one-way analysis of variance (ANOVA) followed by post hoc analysis (Bonferroni test) was used in data that passed both normality and equal variance; otherwise, a non-parametric Kruskal–Wallis test followed by Dunn’s post hoc analysis was used. Data were considered statistically significant at *p* < 0.05.

## 5. Conclusions

Taken together, our findings point out a functional endothelial role for CAR in limiting leukocyte recruitment. We show here that CAR is expressed in human endothelial cells and its activation results in inhibition of leukocyte–endothelial cell interactions under flow, in both in vitro and in vivo. The anti-inflammatory properties of CAR agonism on endothelial cell reactivity are associated with inhibitory effects on adhesion molecule expression and chemokine release. CAR is, therefore, a possible potential therapeutic target for many cardiovascular and metabolic disorders in which aberrant endothelial activation plays a significant pathophysiological role, such as atherosclerosis and diabetes.

## Data Availability

The data presented in this study are available on request from the corresponding author.

## Abbreviations

CAR	Constitutive androstane receptor
TNF	Tumoral necrosis factor
MCP-1	Monocyte chemoattractant protein-1
RANTES	Regulated upon Activation, Normal T cell Expressed and presumably Secreted
HUVEC	Human Umbilical Vein Endothelial Cells
VCAM-1	Vascular cell adhesion molecule 1
MAPK	Mitogen activated protein kinase
NF-κb	Transcription nuclear factor kappa B
